# Systematic evaluation of predictive models for futile recanalization before thrombectomy in patients with acute ischemic stroke

**DOI:** 10.3389/fneur.2025.1625236

**Published:** 2025-10-23

**Authors:** Cheng Chen, Lei Liu, Xiaoling Liu, Ya Tan

**Affiliations:** ^1^Department of Pain, Suining Central Hospital, Suining, Sichuan, China; ^2^Department of Gastroenterology, West China Fourth Hospital, Sichuan University, Chengdu, Sichuan, China; ^3^Department of Rehabilitation, Suining First People's Hospital, Suining, Sichuan, China; ^4^Department of Geriatrics, Suining Central Hospital, Suining, Sichuan, China

**Keywords:** acute ischemic stroke, large vessel occlusion, mechanical thrombectomy, futile recanalization, predictive model, systematic review

## Abstract

**Objective:**

To systematically review existing predictive models for futile recanalization after mechanical thrombectomy in patients with acute ischemic stroke, in order to provide a basis for treatment decision-making.

**Methods:**

Relevant studies on predictive models of futile recanalization after mechanical thrombectomy for acute ischemic stroke were searched in PubMed, Web of Science, Embase, The Cochrane Library, CNKI, Wanfang, and VIP databases from inception to May 5, 2024. Reference lists were also manually searched as supplements. Two researchers independently performed the literature search, screening, and data extraction, and conducted risk of bias and quality assessments. Because most included studies did not provide 95% confidence intervals or standard errors of AUC values, a formal quantitative meta-analysis of model performance was not feasible. Instead, we conducted a stratified descriptive synthesis of AUC values according to modeling approach (traditional regression vs. machine learning/deep learning).

**Results:**

Thirteen studies were included, encompassing 23 predictive models for futile recanalization. Variables used in the models mainly involved baseline clinical and imaging features. The most frequently included predictors were age, NIHSS score, baseline mRS score, and baseline Alberta Stroke Program Early CT Score (ASPECTS). The AUC of the models ranged from 0.650 to 0.981, with 11 models reporting AUC values ≥0.8, indicating high predictive performance.

**Conclusion:**

Predictive models for futile recanalization after mechanical thrombectomy in acute ischemic stroke are still under development. While many models exhibit good discrimination, they commonly face a high risk of bias. Future research should emphasize external validation and optimization of existing models to improve their performance, reduce bias, and promote clinical implementation.

**Systematic review registration:**

The systematic review was registered in PROSPERO under the ID CRD42022382797. https://www.crd.york.ac.uk/prospero/display_record.php?ID=CRD42022382797.

## 1 Introduction

Stroke is the leading cause of disability and the second leading cause of death globally, with ischemic stroke accounting for approximately 82% of all cases. Data indicate that deaths due to ischemic stroke have increased by 60.68% over the past 30 years. With the aging population trend intensifying, the burden of ischemic stroke is expected to rise further (1, 2). National and international guidelines recommend mechanical thrombectomy (MT) as an effective treatment for acute large vessel occlusion (LVO) ischemic stroke, suitable within 24 h of onset for patients with anterior circulation occlusion and salvageable brain tissue (3). Despite the widespread adoption and continuous optimization of MT, futile recanalization remains a significant clinical issue, with postoperative rates ranging from 49 to 67% (4). Futile recanalization refers to achieving mTICI grade 2b or 3 recanalization after endovascular therapy without attaining functional independence at 90 days (5).

Numerous predictive models have been developed and validated to assess the risk of futile recanalization post-MT in patients with acute ischemic stroke, such as the HIAT and THRIVE scores (6, 7). These models, like those predicting symptomatic intracranial hemorrhage (sICH) after intravenous thrombolysis (e.g., MSS, HAT, SITS-sICH, GRASPS, SPAN-100, and SEDAN), are mainly based on traditional logistic regression (LR) methods. However, LR-based models are prone to issues such as multicollinearity and overfitting (5).

In recent years, with a deeper understanding of stroke pathophysiology, more factors such as patient history, laboratory parameters, and imaging characteristics have been found to be associated with futile recanalization after MT. Machine learning (ML) algorithms have shown strong utility in stroke diagnosis, treatment, and prognosis prediction, leading to the development of many new models. Nonetheless, current models vary significantly in quality, performance, and clinical applicability, and systematic reviews are lacking. Therefore, this study aims to synthesize and evaluate existing predictive models of futile recanalization through systematic review, quality assessment, and meta-analysis to provide scientific evidence for model optimization and clinical application.

## 2 Methods

This systematic review was registered in PROSPERO (ID: CRD42022382797) and was conducted in accordance with the PRISMA guidelines.

### 2.1 Search strategy

A comprehensive search was conducted in PubMed, Web of Science, Embase, The Cochrane Library, CNKI, Wanfang, and VIP databases for studies on predictive models of futile recanalization after mechanical thrombectomy in patients with acute ischemic stroke. The search period spanned from database inception to May 5, 2024. Additional references were identified through manual screening of bibliographies. Chinese search terms included: “ischemic stroke/stroke/cerebrovascular accident/cerebral infarction/predictive model/endovascular recanalization/mechanical thrombectomy”; English search terms included: “ischemic stroke”/“brain ischemia”/“large vessel occlusion”/“endovascular thrombectomy”/“mechanical thrombectomy”/“risk prediction model.”

### 2.2 Inclusion and exclusion criteria

#### 2.2.1 Inclusion criteria

(1) Study types: case-control and cohort studies; (2) Study population: patients aged ≥8 years with a diagnosis of stroke based on commonly accepted criteria and confirmed by CT or MRI; (3) Content: development and/or validation of predictive models for futile recanalization.

#### 2.2.2 Exclusion criteria

(1) Models including non-LVO stroke patients (e.g., hemorrhagic or lacunar strokes); (2) Duplicate publications from the same cohort; (3) Studies with incomplete model construction information or lacking performance assessment; (4) Conference abstracts, reviews, letters, commentaries, editorials, and corrigenda were excluded.

### 2.3 Literature screening

After de-duplication in EndNote X9, two reviewers independently screened the studies based on inclusion and exclusion criteria. Title and abstract were screened initially, followed by full-text evaluation. Disagreements were resolved through discussion or consultation with a third reviewer.

### 2.4 Data extraction

Data were extracted by two researchers according to the CHARMS checklist (Critical Appraisal and Data Extraction for Systematic Reviews of Prediction Modeling Studies) (8). A standardized form was used to ensure consistency, capturing details such as first author, publication year, title, country, study design, sample size, data source, diagnostic methods, number of models, outcome indicators, candidate predictors, modeling methods, variable selection techniques, model performance, validation methods, model presentation, and number and names of predictors.

### 2.5 Risk of bias and quality assessment

Risk of bias was assessed using the Prediction Model Risk of Bias Assessment Tool (PROBAST) (9), which includes 20 items across four domains: participants, predictors, outcomes, and analysis. Two researchers independently evaluated the studies, and discrepancies were resolved by a third reviewer. The Transparent Reporting of a Multivariable Prediction Model for Individual Prognosis or Diagnosis (TRIPOD) checklist was used to assess reporting quality.

### 2.6 Statistical analysis

Model performance was assessed using the area under the receiver operating characteristic curve (AUC), with corresponding 95% confidence intervals (CI) extracted. Descriptive statistics were used to summarize study characteristics, model development and validation, and performance metrics.

## 3 Results

### 3.1 Literature search results

**Figure 1:**
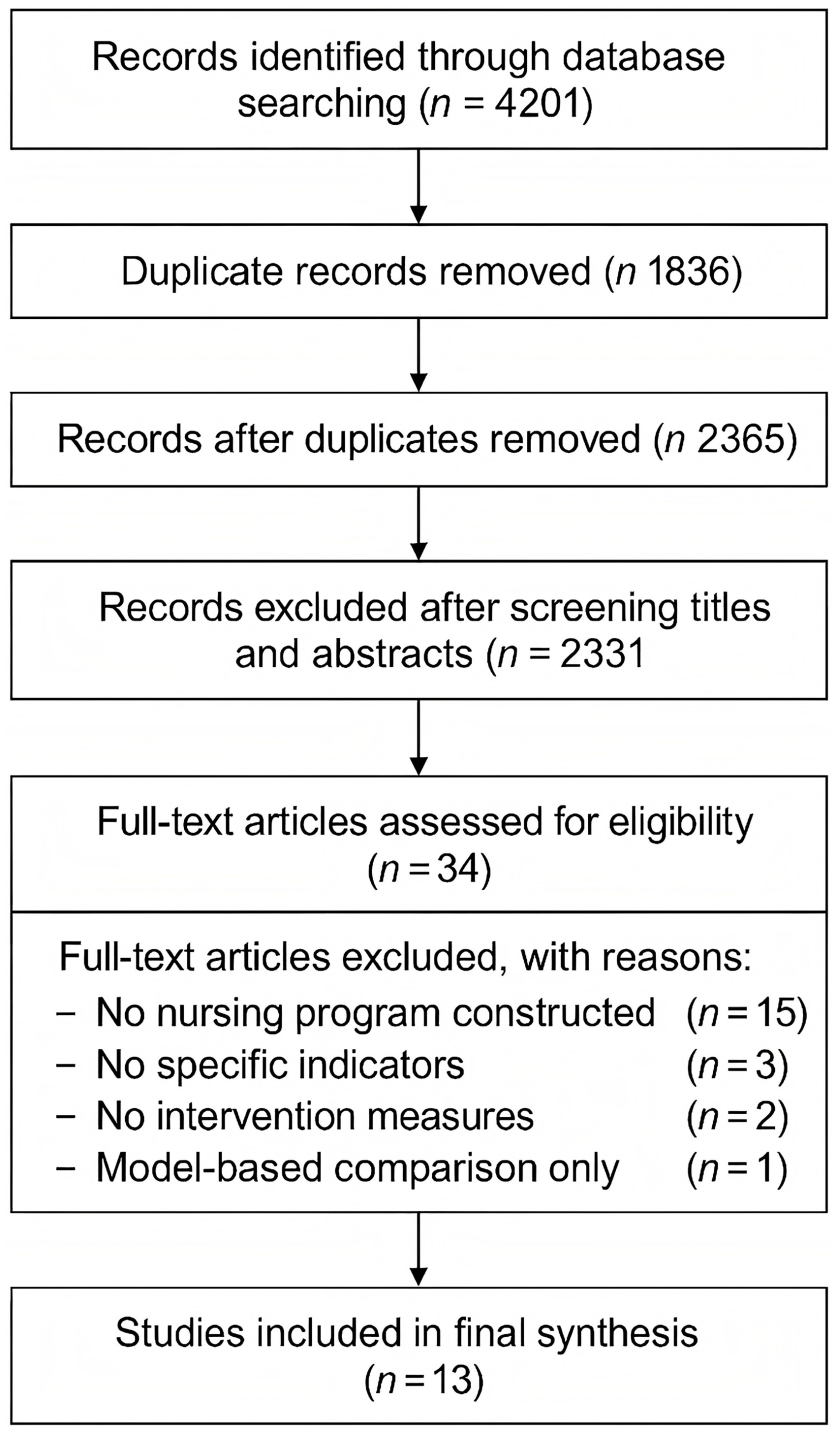
PRISMA flow diagram of the literature screening and selection process for included studies.

A total of 4,201 studies were initially identified. After removing duplicates, 2,365 articles remained. Following title and abstract screening and full-text review according to inclusion and exclusion criteria, 13 studies were included in the final analysis. These studies reported 23 predictive models for futile recanalization (10–22). The study selection process is illustrated in Figure 1.

### 3.2 Basic characteristics of included studies

The 13 studies were published between 2014 and 2023, including 2 in Chinese (20, 22) and 11 in English (10–19, 21). Seven studies were multicenter (11–15, 17, 20), and six were single-center studies (10, 16, 18, 19, 21, 22). Four studies developed four models each (11, 14, 17, 19), while the remaining nine developed a single model. Detailed characteristics of the included studies are presented in Table 1.

**Table 1:** Basic characteristics of included studies.

Study	Diagnostic method	Variable selection method	Key features	Sample size	
				All	Ineffective recanalization
Brugnara et al. (10)	NCCT and CTA	–	Baseline mRS, baseline infarct volume, NIHSS, time from symptom onset to imaging, baseline ASPECTS	246	165
Feyen et al. (11)	CT or MRA	Machine learning algorithm	NIHSS, age, baseline mRS	1,138	615
Grech et al. (12)	CT	Multivariate analysis	Age, admission NIHSS, leptomeningeal collaterals	55	26
Hu et al. (13)	–	Multivariate analysis	NIHSS, creatinine, puncture-to-recanalization time, LDL, diastolic BP, platelets, fasting glucose, TOAST type	238	156
Jabal et al. (14)	CT and CTA	Shapley explainability analysis	Age, baseline NIHSS, brain atrophy, occlusion side, ASPECTS, collateral defect volume	293	192
Li et al. (15)	CT	Multivariate analysis	NIHSS, creatinine, age	238	157
Lin et al. (16)	CT or MRI	Multivariate analysis	Stroke history, admission NIHSS, ASPECTS	84	42
Nishi et al. (17)	CT	Multivariate analysis	LR: Care dependence, Occlusion site, Sex, Atrial fibrillation, mRS RLR: Care dependence, Age, mRS score, ASPECTS score, NIHSS score SVM: Age, ASPECTS score, NIHSS score, Intravenous thrombolysis with Tpa RF: Age, NIHSS score, mRS score, ASPECTS score, care dependence	115	72
van Walderveen et al. (18)	CT	Logistic, LASSO, Elastic Net, RF	Admission blood pressure, time from stroke onset to groin puncture, platelets, age, creatinine, C-reactive protein, baseline NIHSS score, Thrombus Burden Score, Glasgow Coma Score, baseline ASPECTS score, blood glucose, site, history of atrial fibrillation	1,383	858
Zeng et al. (19)	NCCT and CT	Shapley explainability analysis	Vascular territory, baseline NIHSS, max hyperdense area	110	61
Chen et al. (20)	MRI	LASSO	Imaging features	45	22
Hilbert et al. (21)	CTA	SDAE	Imaging features	1,301	463
Wei et al. (21, 22)	MRI	LASSO	Age, admission NIHSS, infarct volume	147	147

NCCT, non-contrast CT; CTA, computed tomography angiography; mRS, modified Rankin scale; NIHSS, National Institute of Health stroke scale; ASPECTS, Alberta stroke program early CT score; stacked denoising convolutional self-encoding. NIHSS, National Institute of Health stroke scale; ASPECTS, Alberta stroke program early CT score, stacked denoising convolutional autoencoder (stacked), and stacked stroke program early CT score (ASPECTS). Denoising convolutional auto-encoders (SDAE); Tpa, tissue plasminogen activator.

### 3.3 Model development

The sample sizes in the included studies ranged from 45 to 1,383 participants. The primary modeling approaches included logistic regression and machine learning algorithms (see Table 2). Six studies employed univariate analysis for variable selection (12, 13, 15–18, 21); three studies applied the least absolute shrinkage and selection operator (LASSO) (18, 19, 22); and five studies used machine learning-based selection methods (11, 14, 18, 19, 21). One study did not specify the method for variable selection (10). When stratified by modeling approach, regression-based models (*n* = 4) achieved consistent performance, with AUCs ranging from 0.78 to 0.87 (mean 0.83, median 0.84). In contrast, machine learning/deep learning models (*n* = 18) exhibited a wider distribution of performance (0.65–0.98), with a mean AUC of 0.81 and a median of 0.78 (Table 3).

**Table 2:** Model performance and validation.

Study	Model	AUC		Other metrics	Internal validation	External validation	Presentation
		Train	Test				
Brugnara et al. (10)	AdaBoost	0.740	–	ACC: 0.711	Bootstrapping	–	–
Feyen et al. (11)	RF	0.76	0.74	SEN: 0.52, SPE: 0.85, ACC: 0.72	10-fold cross validation	–	–
	SVW	0.75	0.48	SEN: 0.45, SPE: 0.87, ACC: 0.71			
	KNN	0.73	0.72	SEN: 0.51, SPE: 0.78, ACC: 0.68			
	NNET	0.76	0.77	SEN: 0.5, SPE: 0.83, ACC: 0.71			
	GLM	0.76	0.75	SEN: 0.50, PE: 0.85 ACC: 0.71			
Grech et al. (12)	LR			ACC: 0.76			Formula
Hu et al. (13)	XGBoost	0.835		ACC: 0.75	10-fold cross validation		
Jabal et al. (14)	XGBoost	0.83		ACC: 0.74	10-fold cross validation		
	RF	0.76		ACC: 0.76			
	KNN	0.79		ACC: 0.79			
	GB	0.68		ACC: 0.62			
Li et al. (15)	LR	0.816		SEN: 0.48, SPE: 0.92 NPV: 0.48, PPV: 0.91	Bootstrapping		Nomogram
Lin et al. (16)	LR	0.866					Dynamic nomogram
Nishi et al. (17)	LR	0.78 ± 0.08	0.56 ± 0.07		10-fold cross validation	Yes	
	RLR	0.86 ± 0.05	0.90 ± 0.02		10-fold cross validation	Yes	
	SVM	0.86 ± 0.06	0.89 ± 0.01		10-fold cross validation	Yes	
	RF	0.85 ± 0.07	0.87 ± 0.01		10-fold cross validation	Yes	
van Walderveen et al. (18)	SL	0.90			10-fold cross validation		
Zeng et al. (19)	LR- stacking model	0.949		SEN: 0.882 SPE: 0.875 ACC: 0.879	10-fold cross validation		
Chen et al. (20)	SVM	0.981		SEN: 0.944 SPE: 0.941 ACC: 0.943 External Validation: SEN: 0.864 SPE: 0.783 ACC: 0.822	Five-fold cross validation	是	
Hilbert et al. (21)	DL	0.65			Four-fold cross validation		
Wei et al. (22)	SVW	0.925		SEN: 100%, SPE: 75%			Nomogram

AdaBoost, adaptive boosting; RF, random forest; SVM, support vector machine; KNN, *K*-nearest neighbors; NNET, neural network; GLM, generalized linear model; LR, logistic regression; EGR, extreme gradient boosting; NNET, neural network; XGBoost, extreme gradient boosting; GB, gradient boosting; RLR, regularized logistic regression; DL, deep learning; ACC, accuracy; SEN, sensitivity/recall; SPE, specificity; NPV, negative predictive value; PPV, positive predictive value.

**Table 3:** Summary of model performance stratified by modeling approach.

Modeling approach	Number of models (*n*)	Mean AUC	Median AUC	Range of AUC
Traditional statistical models	4	0.83	0.84	0.78–0.87
Machine learning/deep learning models	18	0.81	0.78	0.65–0.98

The AUC values of the 23 models ranged from 0.650 to 0.981, with 11 models achieving AUC ≥ 0.8, indicating good predictive performance. One model was calibrated using the Brier score (13) and another using the Hosmer–Lemeshow test (15); the rest did not report calibration measures. Internal validation was conducted in 11 studies (10, 11, 13–21), while five models underwent both internal and external validation (17, 20). Among the internally validated models, two used bootstrapping (10, 15), while the others employed cross-validation techniques. Three studies presented their models using nomograms (15, 16, 22), enabling intuitive and individualized risk prediction for clinical use. A comparative summary of external validation, calibration reporting, and nomogram availability across the included models is presented in Table 4. Notably, only five models underwent external validation, calibration was reported in only two studies, and three models were presented as nomograms.

**Table 4:** Comparative summary of external validation, calibration reporting, and nomogram availability among included predictive models.

Study/model	External validation	Calibration reported	Nomogram available
Brugnara (AdaBoost)	No	No	No
Feyen (RF/SVM/KNN/NNET/GLM)	No	No	No
Hu (XGBoost)	No	No	No
Jabal (XGBoost/RF/KNN/GB)	No	No	No
Li (LR)	No	Hosmer–Lemeshow	Yes
Lin (LR)	No	No	Yes (dynamic)
Nishi (LR/RLR/SVM/RF)	Yes (partly)	Brier score	No
van Os (SL)	No	No	No
Zeng (stacking LR–ML)	No	No	No
Chen (SVM)	Yes	No	No
Hilbert (DL)	No	No	No
Wei (SVM)	No	No	Yes

### 3.4 Predictive factors included in the models

Across the 23 predictive models, a total of 39 distinct predictors were reported. The most frequently used variables were age, NIHSS score, baseline mRS score, and baseline ASPECTS. These factors were repeatedly identified as key indicators of poor functional outcomes despite successful recanalization. The presentation formats of the models included mathematical formulas and interactive nomograms.

### 3.5 Risk of bias assessment

#### 3.5.1 Participants domain

All 13 studies exhibited a low risk of bias in the participants domain overall. However, two studies were rated as having an unclear risk concerning the appropriateness of inclusion and exclusion criteria (11, 18), as their datasets were derived from multiple cohorts with potentially inconsistent enrollment standards.

#### 3.5.2 Predictors domain

Most studies demonstrated a low risk of bias in the predictors domain. Nonetheless, in eight studies, the consistency of predictor definition and measurement across all participants was unclear (11–15, 17, 20, 22). This was primarily due to the retrospective design of the datasets, where data were not originally collected for the purpose of model development or validation, raising concerns about blinded assessment of predictors.

#### 3.5.3 Outcomes domain

Bias in the outcomes domain was generally low. Some studies, however, lacked detailed descriptions of how outcome variables were defined and whether outcome adjudication was independent of predictor information. This raised potential concerns regarding assessment bias.

#### 3.5.4 Analysis domain

**Figure 2:**
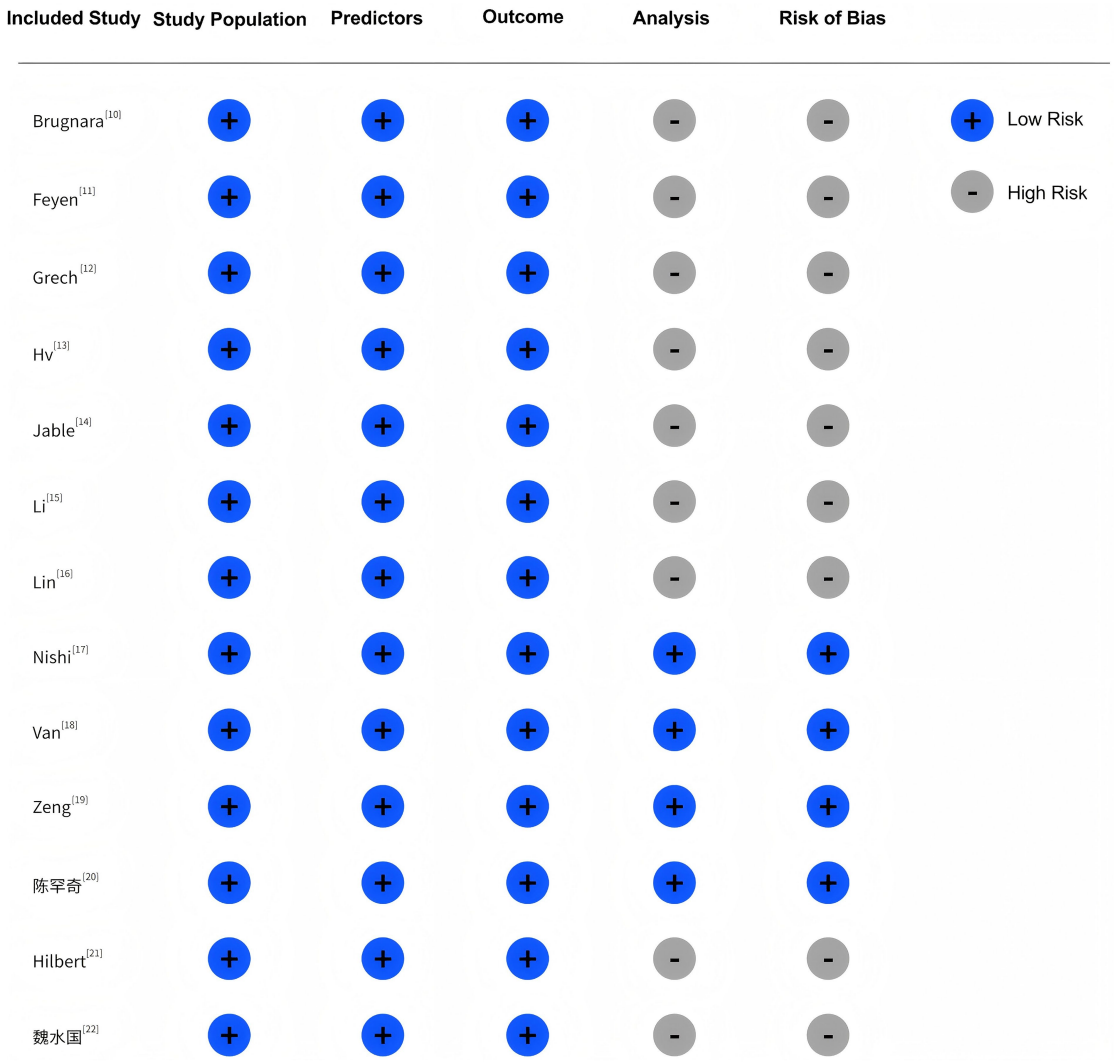
Risk of bias and applicability assessment of included studies.

According to best practices, development studies should include at least 20 events per predictor variable (EPV), and validation studies should enroll at least 100 participants. Five studies did not meet these criteria (12, 14–16, 21). Most studies also did not report how continuous variables were handled, and categorization may have led to information loss. Missing data were managed in six studies through deletion, single imputation, or multiple imputation; the remainder did not report any missing data handling strategies (10, 11, 13, 16, 18, 19). Six studies used univariate analysis for predictor selection (12, 13, 15–18, 21), potentially omitting relevant covariates and increasing bias risk. Detailed risk of bias assessments are shown in Figure 2.

### 3.6 Quality assessment of included studies

According to TRIPOD criteria, all included studies achieved a “good” rating (reporting >70% of required items). However, most studies lacked detailed reporting on sample size calculations, handling of missing data, procedures for model updating, full parameter estimates, application instructions, and updated model results.

## 4 Discussion

### 4.1 General characteristics of predictive models for futile recanalization

**Figure 3:**
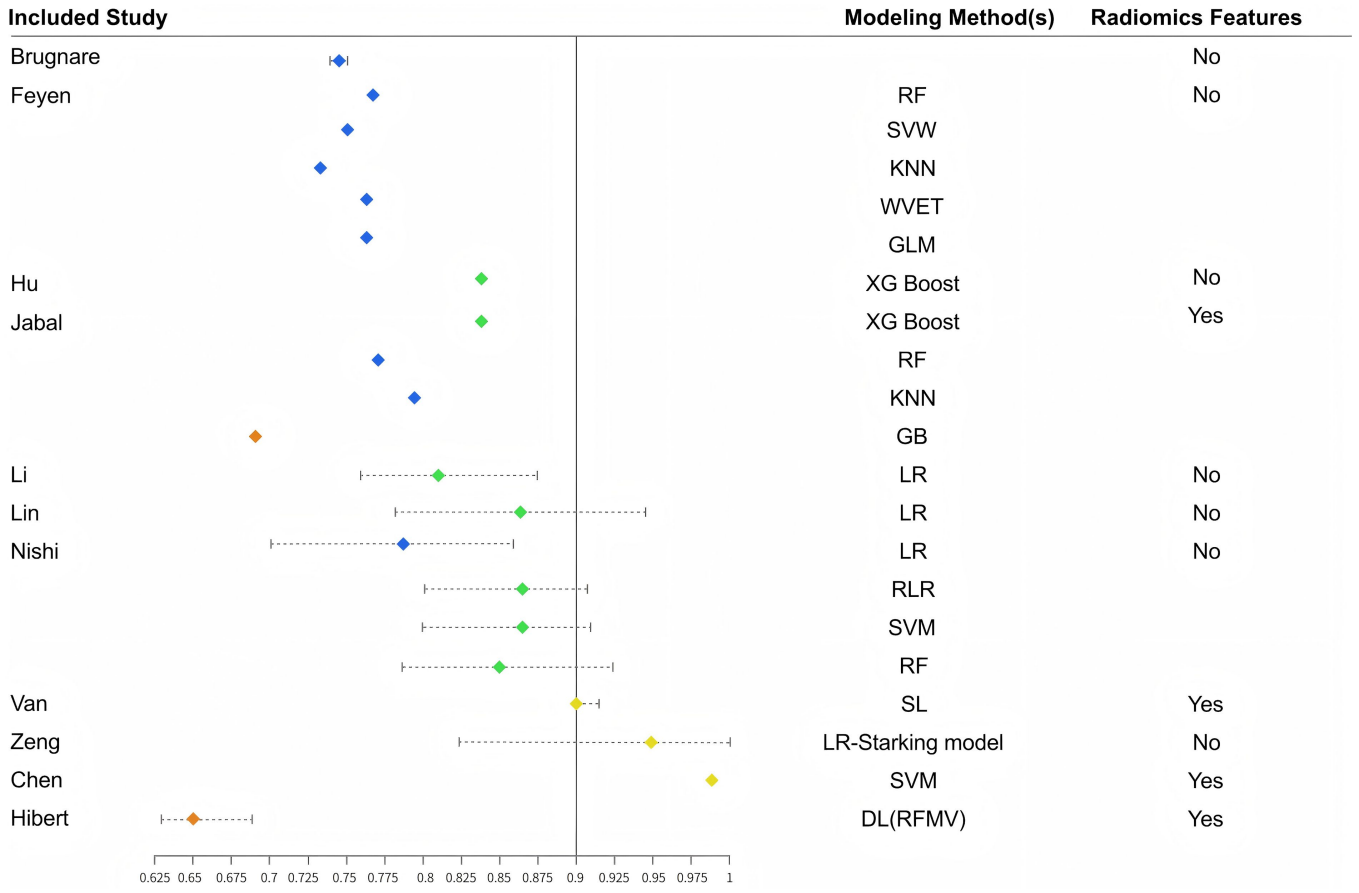
Distribution of AUCs by different model construction methods.

This systematic review comprehensively examined predictive models for futile recanalization following mechanical thrombectomy in patients with acute ischemic stroke. Overall, this research area is still in its developmental phase, with a wide temporal span among the included studies. Nevertheless, the models generally demonstrated good predictive performance. Among the 23 models constructed across 13 studies, AUC values ranged from 0.650 to 0.981. As shown in Figure 3, 20 models had AUC values ≥0.70, and 11 models achieved AUC values ≥0.80, reflecting strong discriminatory ability for identifying patients at risk of futile recanalization.

Internal validation was performed in 20 models, which is critical to reduce overfitting and improve generalizability (23). Several studies utilized nomograms for model visualization, enabling intuitive, individualized risk assessment. Nomograms have become increasingly popular in clinical prediction due to their user-friendly, graphical interface, enhancing clinical applicability. For stroke clinicians and nurses, such tools may support personalized decision-making and optimize perioperative care strategies for patients undergoing mechanical thrombectomy (24).

### 4.2 Limitations of existing models

While the number of predictive models for futile recanalization has increased in recent years, significant methodological limitations persist, particularly in the analysis domain. Common issues included insufficient event-per-variable ratios, inadequate handling of missing data, reliance on univariate analysis for variable selection, and limited reporting of model calibration and performance metrics.

Sample size estimation is crucial in prediction modeling, and underpowered studies can result in unreliable models. Several included studies failed to meet the recommended sample size threshold, which is typically 20 times the number of candidate predictors (25). Furthermore, only a few studies—such as those by Hu, Lin, and Van (13, 16, 18)—appropriately addressed missing data using imputation techniques. Most others either excluded incomplete cases or did not report missing data handling at all, potentially introducing selection bias and reducing model robustness.

Univariate analysis though statistically convenient, is often inadequate for reliable variable selection as it may omit clinically relevant variables that are not statistically significant in isolation (12, 13, 15–18, 26). It also increases the risk of multicollinearity and overfitting (27). Therefore, integrating multiple variable selection strategies, as demonstrated in Van’s study (18), which used univariate regression, LASSO, elastic net, and random forest-based importance ranking, is highly encouraged.

Moreover, many studies did not report AUC values or calibration metrics. Only two models reported calibration statistics. While the AUC reflects a model’s discriminative ability (28), calibration indicates the agreement between predicted probabilities and observed outcomes, which is equally critical for clinical applicability (29). According to the PROBAST assessment, all models were rated as high risk in the analysis domain. This reflects several methodological shortcomings: many studies did not achieve adequate sample sizes or events-per-variable ratios, increasing the likelihood of overfitting; variable selection was often based on univariate analysis alone, which may overlook relevant predictors; missing data were inadequately addressed; continuous variables were sometimes arbitrarily categorized; and calibration measures were rarely reported. Together, these issues explain the consistently high PROBAST risk ratings and highlight the need for stricter adherence to methodological guidelines in future studies. Future model development should adhere to PROBAST guidelines to minimize bias (9), and to TRIPOD reporting standards to ensure transparency and reproducibility (30).

### 4.3 Key predictors of futile recanalization

Meta-analysis identified age, NIHSS score, baseline mRS, and ASPECTS as the most consistent predictors across models. Baseline mRS has long been recognized as a powerful indicator of functional prognosis in ischemic stroke, and models incorporating this variable generally demonstrated better performance (31).

NIHSS, a standardized tool for quantifying neurological deficit, is widely used for initial stroke severity assessment. While higher NIHSS scores may suggest greater benefit from endovascular therapy, they are also associated with increased risk of futile recanalization. However, the optimal cutoff value remains undetermined (32).

Age was incorporated into 16 out of 22 models. Older patients tend to have greater comorbidity burden and poorer functional reserve, which may compromise recovery even after successful recanalization (33). Although current guidelines do not impose age limits on endovascular therapy, outcomes in patients ≥80 years remain debated, making age a critical factor in shared decision-making.

ASPECTS has also been validated as an independent predictor of futile recanalization (34). Originally proposed by Alberta researchers, ASPECTS is a semi-quantitative scoring system used to assess early ischemic changes on non-contrast CT, with higher scores indicating less infarct burden. Previous studies suggest that patients with ASPECTS ≥7 are more likely to benefit from thrombectomy.

Hilbert et al. (21) examined radiological features as predictors, but clinical utility may be limited by inter-institutional variability in imaging protocols and interpretation. Future multicenter studies with standardized imaging assessment are warranted to clarify the role of radiomics in prediction modeling.

### 4.4 Comparison of machine learning and logistic regression

The comparative advantages of machine learning (ML) versus traditional logistic regression (LR) in clinical prediction remain under discussion. LR relies on predefined assumptions and is well-suited for transparent (35), hypothesis-driven modeling. In contrast, ML emphasizes data-driven discovery, excels at handling high-dimensional and non-linear data, and can uncover hidden patterns to enhance prediction accuracy (36). Our stratified descriptive analysis showed that regression-based models achieved a relatively stable mean AUC of 0.83 (range 0.78–0.87), whereas machine learning/deep learning models achieved a similar mean AUC of 0.81 but with a wider range (0.65–0.98). This variability underscores the double-edged nature of ML approaches: while some models achieved excellent discrimination, others underperformed, likely due to small sample sizes and risk of overfitting.

In this review, models built with ML and deep learning techniques demonstrated generally superior or equivalent performance compared to LR models. For instance, Nishi and Van et al. (17, 18) showed that ML slightly outperformed LR in AUC, albeit with modest margins. These findings suggest ML may serve as a useful adjunct in clinical decision support, particularly when dealing with complex feature interactions.

However, most ML models were trained on relatively small datasets with limited external validation, raising concerns about overfitting and generalizability. Moreover, the interpretability of ML models remains a challenge in clinical settings. While LR provides explicit coefficients for each predictor, ML operates as a “black box,” often requiring advanced techniques such as SHAP or LIME to elucidate feature importance.

According to the “No Free Lunch” theorem proposed by Wolpert and Macready (37), no single algorithm universally outperforms others across all problems. Therefore, applying a range of modeling techniques and selecting the most appropriate approach based on data characteristics and clinical context is essential. Despite their potential, ML and DL models exhibit several important limitations. First, most were trained on relatively small datasets with limited external validation, raising concerns about overfitting and variable generalizability. Second, their interpretability remains limited: unlike regression-based models, which provide explicit coefficients for each predictor, ML models often function as “black boxes,” requiring advanced techniques such as SHAP or LIME to explain feature importance. Third, our stratified descriptive summary indicated that although the mean AUC of ML/DL models (0.81) was comparable to regression-based models (0.83), their performance was markedly more variable (0.65–0.98), suggesting unstable generalizability and the risk of overfitting in small or heterogeneous cohorts. A notable example is the Hilbert DL model, which achieved an AUC of only 0.65. This relatively poor performance may be explained by the small training sample size, as deep learning models typically require large amounts of data to extract robust feature representations. In addition, variability in imaging acquisition and preprocessing across centers may have further limited its generalizability. This case illustrates the vulnerability of ML/DL approaches to overfitting and performance instability when applied in data-limited or heterogeneous clinical settings.

### 4.5 Clinical utility of predictive models

The clinical applicability of prediction models for futile recanalization remains limited. Notably, none of the included studies reported decision curve analysis, which precluded a formal assessment of net clinical benefit. Nevertheless, a narrative evaluation of the existing models suggests several potential implications for practice. Models incorporating readily available clinical variables such as age, NIHSS score, and ASPECTS may assist clinicians in identifying patients at high risk of futile recanalization, thereby informing patient selection for mechanical thrombectomy and guiding perioperative management strategies. Such information may also support shared decision-making and risk communication with patients and families.

In addition, nomogram-based models (e.g., Li, Lin, Wei) offer intuitive (15, 16, 22)^,^ graphical representations of individual risk and are particularly suitable for bedside application and integration into electronic health records. These tools may enhance usability in clinical practice, enabling both physicians and nurses to make more personalized treatment and care plans. Future studies should incorporate decision curve analysis and cost-effectiveness evaluation to further establish the net clinical benefit of prediction models and to facilitate their translation into routine clinical workflows.

## 5 Conclusion

In this systematic review, we comprehensively evaluated 13 studies encompassing 23 predictive models aimed at identifying the risk of futile recanalization after mechanical thrombectomy in patients with acute ischemic stroke. While many models demonstrated satisfactory discriminatory performance, with 11 models reporting an AUC ≥ 0.8, several methodological limitations were observed. These included high risk of bias, insufficient sample size, inadequate handling of missing data, and a lack of external validation. Future research should focus on improving methodological rigor through adherence to PROBAST and TRIPOD guidelines, enhancing external validation across diverse populations and clinical settings, and exploring advanced modeling techniques such as interpretable machine learning. Optimized and validated models may ultimately support individualized decision-making and improve post-thrombectomy outcomes in stroke care.

## Data Availability

The datasets presented in this article are not readily available because this study is based on data extracted from previously published studies. No new patient data were collected or analyzed. Therefore, no dataset is available for sharing.
